# High Capacity Reversible Watermarking for Audio by Histogram Shifting and Predicted Error Expansion

**DOI:** 10.1155/2014/656251

**Published:** 2014-05-14

**Authors:** Fei Wang, Zhaoxin Xie, Zuo Chen

**Affiliations:** ^1^School of Information Science and Engineering, Hunan University, Lushan South Rood, Changsha 410082, China; ^2^College of Computer Science and Technology, Zhejiang University, Hangzhou 310027, China; ^3^Chu Kochen Honors College, Zhejiang University, Hangzhou 310058, China

## Abstract

Being reversible, the watermarking information embedded in audio signals can be extracted while the original audio data can achieve lossless recovery. Currently, the few reversible audio watermarking algorithms are confronted with following problems: relatively low SNR (signal-to-noise) of embedded audio; a large amount of auxiliary embedded location information; and the absence of accurate capacity control capability. In this paper, we present a novel reversible audio watermarking scheme based on improved prediction error expansion and histogram shifting. First, we use differential evolution algorithm to optimize prediction coefficients and then apply prediction error expansion to output stego data. Second, in order to reduce location map bits length, we introduced histogram shifting scheme. Meanwhile, the prediction error modification threshold according to a given embedding capacity can be computed by our proposed scheme. Experiments show that this algorithm improves the SNR of embedded audio signals and embedding capacity, drastically reduces location map bits length, and enhances capacity control capability.

## 1. Introduction

Reversible watermarking is a technique that means after watermark information was embedded into the host data, original data can be restored while watermark information is extracted through a series of processes. Because the reversible watermarking schemes can achieve lossless recovery, they can be widely used in military communication, medical image, and digital forensics fields [1–3]. At present, the reversible watermarking technology research mainly concentrates on image [4–6]. Tian [7] proposed an image reversible watermarking algorithm using difference expansion technique. This algorithm first applied an integer wavelet transform on two adjacent pixels in the image and then embedded the watermark data in host pixel by expanding the difference of adjacent pixel. Finally, the algorithm restored pixel data through the inverse integer transform. In [8], a reversible watermarking algorithm was presented using prediction error expansion. First, the algorithm predicted a pixel value according to three pixels adjacent to the current pixel and then extended the difference between prediction and current value to achieve watermark information embedding. The algorithm has higher embedding capacity capability and better quality of embedded image than Tian's scheme. Digital speech is one of the important forms of digital media, but reversible watermarking algorithm for digital audio is relatively less. Reversible audio watermarking schemes mainly face the following problems: (1) audio in WAV format is a floating number ranged from −1 to 1. In order to transform these data, we must map the data to integer space; (2) human audio system is more sensitive than the human visual system, so even slight alteration of audio signal can easily be perceived; (3) the process of audio signal is much trickier than digital image [9]. In [10], Yan and Wang proposed a reversible audio watermarking scheme based on prediction error expansion method. First, three previous samples of the audio sample were multiplied by integer coefficients individually and then the sum of three results was used to predict the current sample. Second, watermarking information was embedded by expanding the difference between the prediction and current amplitude. This algorithm mapped the audio data to integer interval and achieved recovery of original audio signals. In the algorithm, a location map was used to mark the samples without watermark bits. However, the method suffers from lack of embedding capacity due to large auxiliary embedded location information. In [11], a reversible audio data hiding algorithm based on linear prediction and error expansion was present by A. Nishimura. Relative to [10], this paper improved the prediction coefficients using Burg method and obtained a higher SNR and embedding capacity. However, its capacity has the same drawback with [10] and capacity control capability was not considered.

Differential evolution (DE) algorithm is an optimization algorithm based on swarm intelligence. It can simulate the biological natural selection and evolution. This algorithm was first present by Stron and Price in 1995 [12]. DE algorithm can solve multiparameters optimization without depending on the continuity of objective function and the conductivity of the constraints. Meanwhile, this algorithm has good robustness and universality. It is widely used in many fields [13–16].

The application of histogram to reversible watermarking technique first appeared in image field. Ni et al. [17] proposed a reversible watermarking algorithm based on histogram shifting, where watermark is embedded into the histogram bins. A novel watermarking technique has been presented by Thodi and Rodrígez in [18], where location information is dramatically compressed by using histogram shifting technique. To our best knowledge, however, reversible audio watermarking based on histogram technique has not been reported yet. So, we cannot apply the histogram technique of image field to audio data directly.

In this paper, we proposed a novel reversible audio watermarking scheme based on improved prediction error expansion and histogram shifting which combined with the characteristics of digital audio data. Different from using the fixed set of linear prediction coefficients, we propose the optimization model of prediction coefficients and then use the differential optimization algorithm to determine the optimal linear prediction coefficients, so that the overall prediction errors are as small as possible. In addition, according to the characteristics of audio data, we introduced histogram shifting technique which can dramatically reduce the embedding location information. Meanwhile, we present the method which can compute the prediction error modification threshold according to a given embedding capacity. The experiments show that this algorithm can enhance capacity control capability.

The organization of this paper is as follows. In Section 2, we propose prediction error expansion based on differential evolution.  Section 3 describes how information embedding and extraction are done by using histogram shifting and prediction error expansion. The experiment results and analysis are presented in Sections 4 and 5, respectively. The conclusions are in Section 6.

## 2. Prediction Error Expansion Based on Differential Evolution

### 2.1. Differential Evolution Algorithm

Differential evolution algorithm has the advantages of high efficiency, good convergence, and robustness. It can perform well when deal with multiparameter optimization problem, especially when the solution space is stochastic. Differential evolution algorithm can be divided into the following operations.

(1) The mutation: the mutation of DE is driven by the difference between the parent individuals; the mutation of vector *u*_*i*_^*g*^ is as follows:
  (1) $$\begin{array}{c} v_{i}^{g+1}=u_{r1}^{g}+F\left(u_{r2}^{g}-u_{r3}^{g}\right),\;r_{1}\neq r_{2}\neq r_{3}\neq i, \end{array}$$
  where *r*_1_, *r*_2_, *r*_3_ are uniform random integers ∈[1, *N*_*p*_], *F* ∈ [0,2] represents the control parameters, and *g* means the index of generation.
(2) Crossover: in the DE algorithm, the crossover operation guarantees the diversity of population. When applying crossover on mutant vector *v*_*i*_^*g*+1^ and current individual *u*_*i*_^*g*^, we can get new vector *w*_*i*,*j*_^*g*+1^. The formula of crossover operation is as follows:
  (2) $$\begin{array}{c} w_{i,j}^{g+1}=\{\begin{array}{cc} v_{i,j}^{g+1}, & \left(\text{rand}\left(\right)\leq C_{R}\;\text{or}\;j=l_{i}\right)\\ u_{i,j}^{g}, & \text{otherwise}, \end{array} \end{array}$$
  where *j* ∈ (1,2,…, *D*) and *D* is the dimension of the problem, rand  () means the random values ∈[0,1], and *C*_*R*_ represents the probability of crossover operation. *l*_*i*_ ∈ [1,2,…*D*] is a randomly chosen value.
(3) Selection: the selection operation of DE algorithm is based on a greedy selection scheme. We choose the individual which has better fitness between the vector *w*_*i*_^*g*+1^ and the individual of original population *u*_*i*_^*g*^. The formula is described as follows:
  (3) $$\begin{array}{c} u_{i}^{g+1}=\{\begin{array}{cc} w_{i}^{g+1}, & f\left(w_{i}^{g+1}\right)\leq f\left(u_{i}^{g}\right)\\ u^{g}, & \text{otherwise}, \end{array} \end{array}$$

where *f*(·) represents fitness function.

### 2.2. The Optimization of Linear Prediction Coefficients Based on DE Algorithm

**Figure 1:**
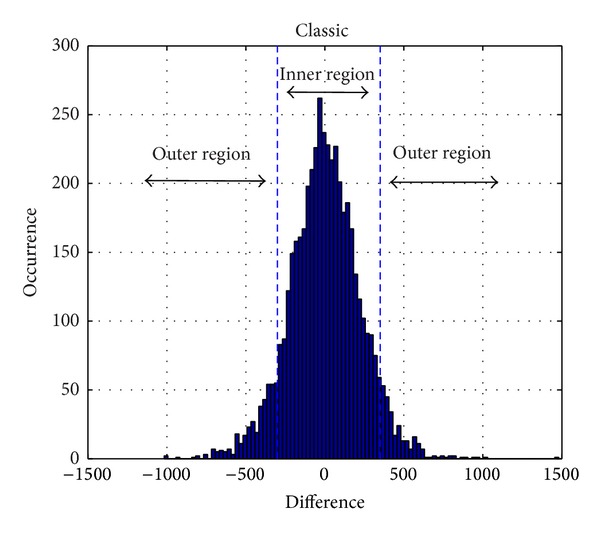
Histogram of prediction errors for classic music.

Figure 1 presents a typical histogram of prediction errors for classic music. The prediction errors histogram for most natural audio would be similar to this. Consider a process of expansion embedding. The smaller the magnitude of the prediction errors, the smaller the distortion of the audio. In the sense, the prediction errors occur more frequently in the central region. Since embedding capacity and distortion performance are most important indicators for reversible audio watermarking scheme, the optimization aim is to maximize SNR of embedded audio and capacity. The formula is presented as follows:

(4) $$\begin{array}{c} F=10\;\text{lo}{\text{g}}_{10}\left(\frac{\sum_{i=1}^{L}S^{2}\left(i\right)}{\sum_{i=1}^{L}\left[S^{2}\left(i\right)-S^{\prime 2}\left(i\right)\right]}\right)\times \frac{l}{L}, \end{array}$$

where *S* ^2^(*i*) and *S* ^′2^(*i*) mean original and embedded audio, respectively, *L* is the length of the audio data, and *l* means the number of embedded bits.

## 3. Prediction Error Expansion Based on Histogram Shifting

### 3.1. Histogram Shifting

**Figure 2:**
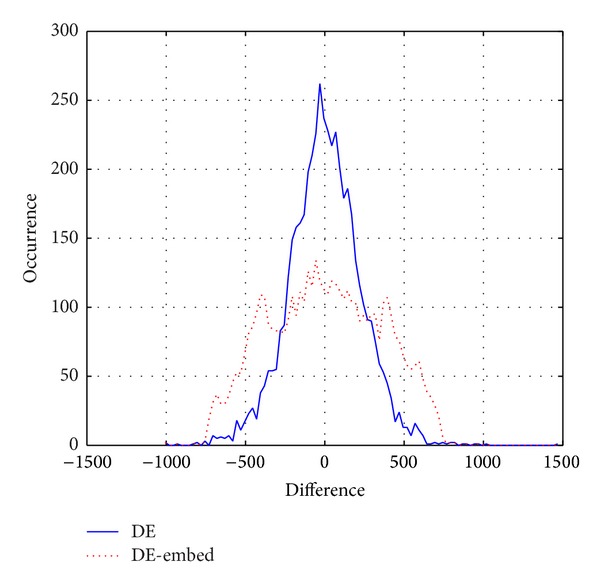
Histogram after expansion of inner region and original prediction errors.

In [18], histogram of prediction error for a host data is divided into two nonoverlapping regions, the outer and the inner regions, which are shown in Figure 1. Expansion of the prediction errors expands the histogram of the inner region. As is shown in Figure 2, after a process of expansion, the peak of the histogram has fallen by almost 50%. Because the histogram of prediction errors for audio does not strictly satisfy the continuity, the algorithm in [18], in which the determination of embedded location was based on symmetrical histogram, cannot be applied to audio reversible watermarking algorithm. In this paper, we assume two thresholds *T*_*l*_ and *T*_*r*_, which can be used to control the boundaries of the inner region and outer region. We first set *T*_*l*_ = 0, *T*_*r*_ = 1. Given a capacity, we can determine the boundaries of inner region and outer region according to the Algorithm 1.

Where *D* means all the prediction errors, function num  (·) returns the index of the given value. In the process of determining the thresholds, we considered the cumulative impact by all expansive bins rather than only the current expansive bin.

### 3.2. Embedding and Extraction

**Figure 3:**
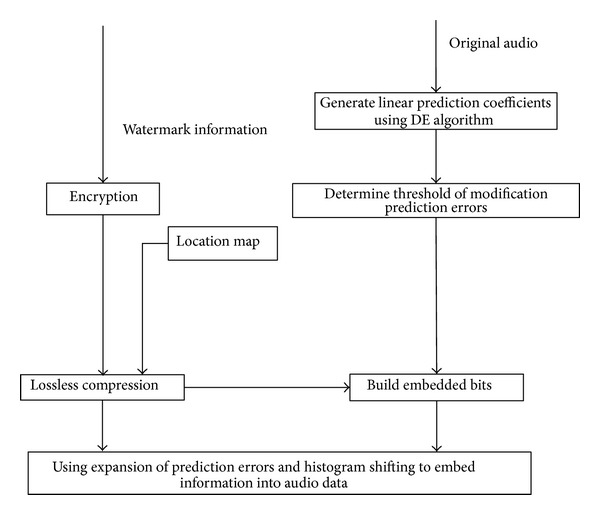
Diagram of watermark embedding process.

All steps of the watermark embedding procedure based on improved prediction error expansion and histogram shifting are shown in Figure 3. The details are described as follows.

**Step 1**

Assume a host audio data *A* = {*a*_1_, *a*_2_, *a*_3_,…, *a*_*N*_ | *a*_*i*_ ∈ [−1,1]}. For audio sample *a*_*i*_, we define *a*_*i*−3_, *a*_*i*−2_, *a*_*i*−1_ as its neighborhood samples and *a*_*i*_′ as its predicted value. As in formula (5), *F*(·) represents predictor:

(5) $$\begin{array}{c} a_{i}^{\prime }=F\left(a_{i-3},a_{i-2},a_{i-1}\right). \end{array}$$

**Step 2**

Define *p*_*i*_ as prediction error *p*_*i*_ = *a*_*i*_ − *a*_*i*_′; then we embedded watermarking bit *b* by expansion of prediction error *p*_*i*_. The formula is as follows:

(6) $$\begin{array}{c} p_{i}^{\prime }=2\times p_{i}+b. \end{array}$$

**Step 3**

Determine the linear prediction coefficients based on DE algorithm. We can define *F*(·) = round(*c*_1_ × *a*_*i*−3_ + *c*_2_ × *a*_*i*−2_ + *c*_3_ × *a*_*i*−1_)  (*i* > 3). According to the optimization scheme proposed in Section 2, we can obtain optimal prediction coefficients *C* = [*c*_1best_, *c*_2best_, *c*_3best_].

**Step 4**

Give capacity *P*. We can obtain threshold *T*_*l*_, *T*_*r*_ based on Algorithm 1.

**Step 5**

Embed watermarking information using histogram shifting and expansion of prediction errors. *T*_eml_ and *T*_emr_ are left and right boundaries of histogram. We can obtain inner region [*T*_*l*_, *T*_*r*_] and outer regions [*T*_eml_, *T*_*l*_)∪(*T*_*r*_, *T*_emr_]. Finally, embed the watermarking information using expansion of prediction errors or achieve location marking using histogram shifting. The formula is described as follows:

(7) $$\begin{array}{c} p_{i}^{\prime }=\{\begin{array}{cc} p_{i}-\text{abs}\left(T_{l}\right) & \text{if}\;p_{i}\in \left[T_{\text{eml}},T_{l}\right)\\ 2\times p_{i}+b & \text{else}\;\text{if}\;p_{i}\in \left[T_{\text{eml}},T_{\text{emr}}\right]\\ p_{i}+\text{abs}\left(T_{r}\right)+1 & \text{otherwise}. \end{array} \end{array}$$

**Step 6**

Build location map. Since *a*_*i*_ ∈ [32767, −32768], in order to prevent overflow and underflow problems during embedding process, we assign a value “1” in location map when the above situations occur. Otherwise we assign a value “0”. The location map should be lossless compressed. We use run-length coding and Huffman coding scheme.

In the process of decoding, we can extract the watermark information and restore host audio data. The details are as follows.

**Step 1**

The current audio sample is obtained from (5) by using restored host data *a*(*i*)  (*i* > 3). If the value of location map is “1,” keep the audio sample unchanged. For prediction errors ranges in [2*T*_*l*_, 2*T*_*r*_ + 1], we can extract the watermark bit and restore audio sample using

(8) $$\begin{array}{c} b=\mathrm{mod}\left(p_{i}^{\prime },2\right),\\ p_{i}=\left\lfloor \frac{p_{i}^{\prime }-b}{2}\right\rfloor ,\\ a_{i}=a_{i}^{\prime }-p_{i}. \end{array}$$

**Step 2**

For histogram shifting, we can restore the audio data by the following formula:

(9) $$\begin{array}{c} p_{i}=\{\begin{array}{cc} p_{i}^{\prime }+\text{abs}\left(T_{l}\right) & \text{if}\;\;p_{i}^{\prime }<2\times T_{l}\\ p_{i}^{\prime }-\text{abs}\left(T_{r}\right)-1 & \text{if}\;\;p_{i}^{\prime }>2\times T_{r}+1. \end{array} \end{array}$$

## 4. Experiment Set

In this section, we choose five common audio types to demonstrate the performance of the proposed algorithm. All audio files are 16-bit mono in wave format and the sampling rates of them are 44.1 kHz. The detailed description is listed in Table 1. For optimization of linear prediction coefficients based on DE algorithm, we set the size of initial population to 65. The iteration is set to 50. The probability of crossover and mutation are 0.9 and 0.09, respectively.

### 4.1. Evaluation Method

In order to evaluate the performance of the proposed algorithm, we choose two common indicators SNR and capacity. SNR is a statistical difference metric to measure the perceptual similarity between original audio and embedded audio. The formula is as follows:

(10) $$\begin{array}{c} \text{SNR}\left(S,S^{\prime }\right)=10\;\text{lo}{\text{g}}_{10}\left(\frac{\sum_{i=1}^{L}S^{2}\left(i\right)}{\sum_{i=1}^{L}{\left[S\left(i\right)-S^{\prime }\left(i\right)\right]}^{2}}\right), \end{array}$$

where *S*(*i*), *S*′(*i*) are original audio sample and embedded audio sample, respectively. Capacity is used to measure how much information can be embedded into a host data. The formula is as follows: where *N*_*L*_ means the required number of audio sample when embedding one bit watermark during embedding procedure

(11) $$\begin{array}{c} p=\frac{1}{N_{L}}. \end{array}$$

## 5. The Experimental Results and Analysis

**Figure 4:**
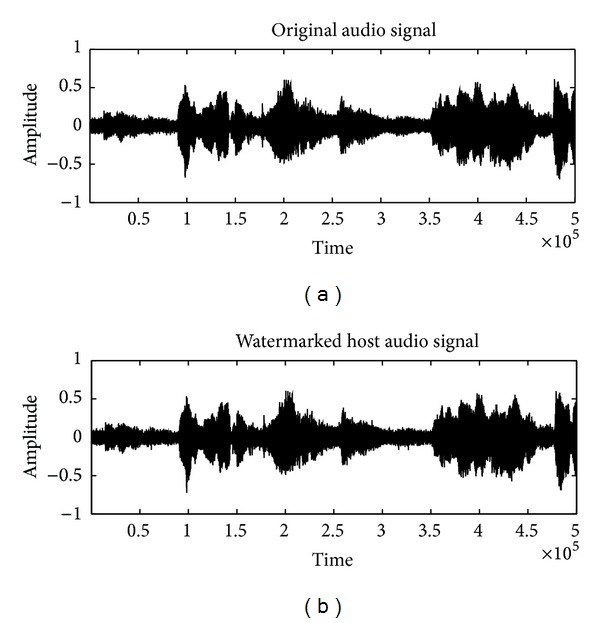
Original pop audio and the watermarked audio signal.

**Figure 5:**
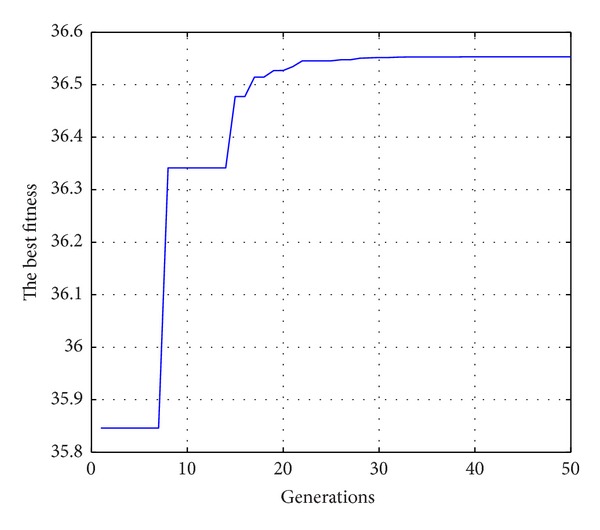
Convergence curves of object function with DE algorithm.

**Figure 6:**
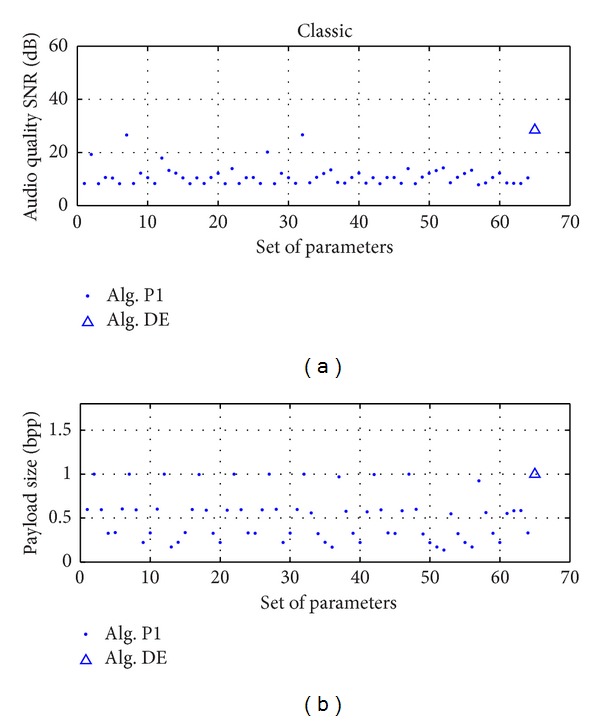
The performance of the 64 prediction coefficient sets and optimal prediction coefficient set for classic audio.

Using the ED algorithm settings in Section 4, the convergence curves of object function with DE algorithm are shown in Figure 5. The best fitness of an individual can converge fast to a stable value in the twenty-fifth generation. The comparison of SNR and capacity between embedded audio using the optimal linear prediction coefficients and best linear prediction coefficients proposed in [10] was shown in Table 2. We can see that the linear prediction coefficients generated by DE algorithm achieve better SNR and capacity than fixed coefficients for all the types of test audios in the same condition.  Figure 6 presents the performance of the 64 prediction coefficient sets and optimal prediction coefficient set for classic audio. It can be seen that linear prediction coefficients obtained by DE have obvious advantage in SNR and capacity. The original and watermarked audio signals in time domain are shown in Figure 4; the difference between them is invisible.

**Figure 7:**
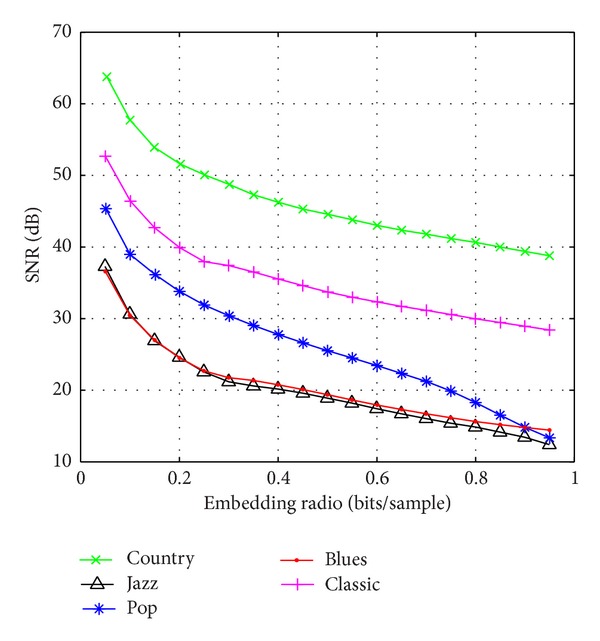
The embedding capacity versus the embedded.

**Figure 8:**
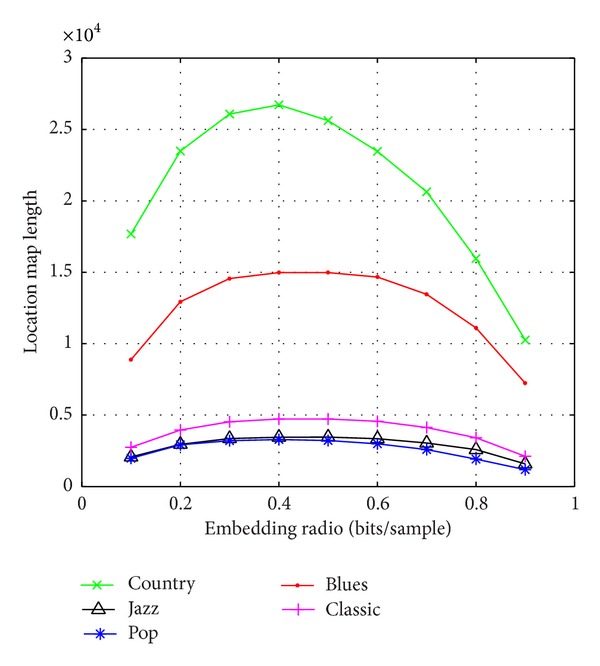
The embedding capacity versus compressed location map length curve of five types audio quality curve of five types audio.

Figure 7 graphically depicts the embedding capacity versus the embedded audio quality curve of five types of audio. We can see that the SNR of embedded audio decreased with the increase of embedding capacity. According to the given embedding capacity, we can conveniently determine and modify the threshold of prediction error. That enhances capacity control capability. In order to evaluate the performance of proposed algorithm based on histogram shifting, we use run-length coding and Huffman coding scheme to compress the location map.  Figure 8 presents the embedding capacity versus compressed location map length curve of five types of audio. The location map is generated using algorithm proposed in [11]. We can see that location map of algorithm compared has a large length and the compressibility of location map is low. The large auxiliary location map will take a lot of capacity. In this paper, we adopt histogram shifting to reduce the location map. As we know, we can map the audio data to integer ranged from −32768 to 32767. As the range of audio data is very wide, the overflow phenomenon rarely occurs. Meanwhile, the location map of proposed algorithm only needs overflow location information, so the length of location map can be reduced drastically. The experiments showed that the length of location map using proposed algorithm is negligible.

## 6. Conclusion

This paper proposed a novel reversible audio watermarking algorithm based on improved prediction error expansion and histogram shifting. As the performance of reversible audio scheme using prediction error expansion is affected by the predictor, we proposed the optimization of linear prediction coefficients using differential evolution algorithm. The experiments have shown that the proposed scheme can achieve better embedded audio quality and higher capacity. In order to reduce the length of location map, we introduced histogram shifting scheme. In addition, we proposed the scheme which can be used to compute prediction error modification threshold according to a given embedding capacity. The simulation results verify our scheme can drastically reduce location map bits length and enhance capacity control capability.

## Figures and Tables

**Algorithm 1:**
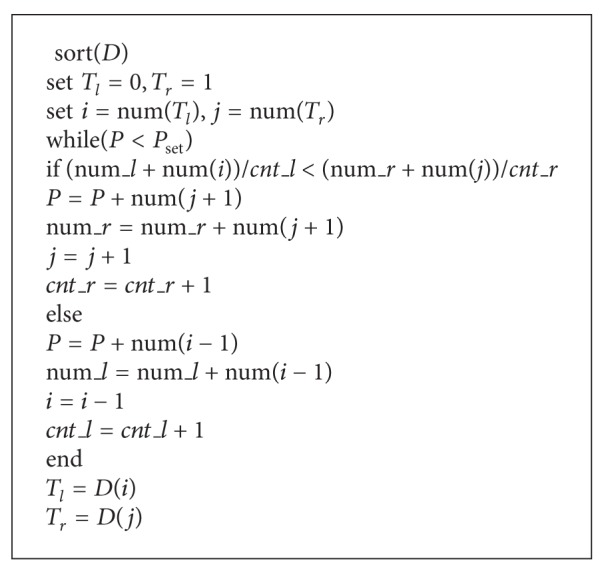


**Table 1:** Test database 16 bits.

Index	Audio description
1	Blues
2	Classic
3	Jazz
4	Pop
5	Country

**Table 2:** The comparison of SNR and capacity between embedded audio using the optimal linear prediction coefficients and best linear prediction coefficients proposed in [10].

Audio	Set *T* = 5000			
	Ours		[10]	
	SNR	Capacity	SNR	Capacity
Blues	38.14	0.99	37.00	0.99
Classic	15.32	0.99	10.06	0.99
Jazz	16.73	0.99	16.07	0.97
Pop	18.13	0.99	15.87	0.99
Country	28.48	0.99	26.64	0.99
